# Cognitive profile, neuroimaging and fluid biomarkers in post-acute COVID-19 syndrome

**DOI:** 10.1038/s41598-024-63071-2

**Published:** 2024-06-05

**Authors:** Núria Guillén, Agnès Pérez-Millan, Neus Falgàs, Gema M. Lledó-Ibáñez, Lorena Rami, Jordi Sarto, Maria A. Botí, Cristina Arnaldos-Pérez, Raquel Ruiz-García, Laura Naranjo, Bàrbara Segura, Mircea Balasa, Roser Sala-Llonch, Albert Lladó, Sarah M. Gray, Jason K. Johannesen, Madeline M. Pantoni, Grant A. Rutledge, Ruta Sawant, Yi Wang, Luke S. Watson, Josep Dalmau, Raquel Sanchez-Valle

**Affiliations:** 1 Alzheimer’s Disease and Other Cognitive Disorders Unit, Service of Neurology, Hospital Clínic de Barcelona, Barcelona, Spain; 2 Fundació Recerca Clínic Barcelona-IDIBAPS, Barcelona, Spain; 3 Institut de Neurociències, Faculty of Medicine and Medical Sciences, University of Barcelona, Barcelona, Spain; 4 Department of Biomedicine, Faculty of Medicine, University of Barcelona, Barcelona, Spain; 5 Department of Autoimmune Diseases, Hospital Clínic, Barcelona, Spain; 6 Immunology Service, Biomedical Diagnostic Center, Hospital Clínic de Barcelona, Barcelona, Spain; 7 Medical Psychology Unit, Department of Medicine, University of Barcelona, Barcelona, Spain; 8 Centro de Investigación Biomédica en Red Sobre Enfermedades Neurodegenerativas (CIBERNED), Madrid, Spain; 9 Centro de Investigación Biomédica en Red de Bioingeniería, Biomateriales y Nanomedicina (CIBER-BBN), Barcelona, Spain; 10 Sage Therapeutics, Cambridge, USA; 11 Department of Neurology, Hospital Clínic de Barcelona, University of Barcelona, Barcelona, Spain; 12 Enfermedades Raras, Centro de Investigación Biomédica en Red, Madrid, Spain; 13 Department of Neurology, Perelman School of Medicine, University of Pennsylvania, Philadelphia, PA USA; 14 Catalan Institute for Research and Advanced Studies (ICREA), Barcelona, Spain

**Keywords:** Post-acute COVID-19, Cognitive symptoms, MRI, Cytokines, Longitudinal study, Cognitive neuroscience, Neuroimmunology

## Abstract

We aimed to characterize the cognitive profile of post-acute COVID-19 syndrome (PACS) patients with cognitive complaints, exploring the influence of biological and psychological factors. Participants with confirmed SARS-CoV-2 infection and cognitive complaints ≥ 8 weeks post-acute phase were included. A comprehensive neuropsychological battery (NPS) and health questionnaires were administered at inclusion and at 1, 3 and 6 months. Blood samples were collected at each visit, MRI scan at baseline and at 6 months, and, optionally, cerebrospinal fluid. Cognitive features were analyzed in relation to clinical, neuroimaging, and biochemical markers at inclusion and follow-up. Forty-nine participants, with a mean time from symptom onset of 10.4 months, showed attention-executive function (69%) and verbal memory (39%) impairment. Apathy (64%), moderate-severe anxiety (57%), and severe fatigue (35%) were prevalent. Visual memory (8%) correlated with total gray matter (GM) and subcortical GM volume. Neuronal damage and inflammation markers were within normal limits. Over time, cognitive test scores, depression, apathy, anxiety scores, MRI indexes, and fluid biomarkers remained stable, although fewer participants (50% vs. 75.5%; p = 0.012) exhibited abnormal cognitive evaluations at follow-up. Altered attention/executive and verbal memory, common in PACS, persisted in most subjects without association with structural abnormalities, elevated cytokines, or neuronal damage markers.

## Introduction

Post-acute COVID-19 syndrome (PACS) is defined by the continuation or development of new symptoms 3 months after the initial SARS-CoV-2 (COVID-19) infection, lasting for at least 2 months with no other explanation. PACS can affect individuals irrespective of the severity of their acute symptoms or their premorbid condition. Notably, non-hospitalized patients with mild acute illness, particularly those aged between 36 and 50 years, represent a significant proportion of COVID-19 cases. Additionally, there is a female predominance observed in PACS cases compared to males.

During the acute phase of COVID-19 infection, a diverse range of neurological complications have been documented, encompassing symptoms such as headache, anosmia, dysgeusia, dizziness, agitation, confusion, impaired level of consciousness, or acute stroke^1^. A recent meta-analysis examining the prevalence of persistent symptoms occurring at 12 weeks or more post-acute COVID-19 infection revealed that approximately 22% of subjects presented subjective or objective cognitive impairment^2^. Among patients with PACS, headache and cognitive complaints emerge as the most prevalent neurological symptoms, although a myriad of other neurological manifestations has been reported. Limited studies have analyzed cognitive impairment in PACS using standard cognitive assessment tools, revealing alterations in attention, executive function, and memory^3–7^. Long-term cognitive complaints are associated with heightened levels of anxiety and depression^8^, as well as diminished quality of life^9^. The comprehensive characterization of long-term cognitive repercussions in PACS patients remains to be fully elucidated.

PACS typically manifests with a range of non-neurological symptoms, including fatigue, dyspnea, joint or chest pain. Notably, a study highlighted fatigue as the most prevalent symptom during both the acute and follow-up phases of COVID-19^10^. Furthermore, research focusing on non-hospitalized post-COVID-19 patients with complex residual symptoms revealed significant challenges in returning to normal function. Despite interventions such as exercise, respiratory, olfactory rehabilitation, cognition/speech therapy and psychological support, one third of patients had not resumed work even 22 months after the initial infection^11^.

Various hypotheses have been proposed to elucidate the pathogenesis of PACS, encompassing persisting reservoirs of SARS-CoV-2 in tissues, immune dysregulation, autoimmunity, or microvascular damage^12,13^. Some studies have reported elevated levels of cytokines and neuronal damage markers in patients with acute COVID-19 infection and neurological symptoms^14^, with further associations noted between elevated cytokines or neuronal damage markers and the development of post-acute sequelae of COVID-19^14–16^. Neuroimaging studies in PACS have yielded disparate findings: while certain analyses have documented reductions in cortical thickness (CTh), gray matter (GM) volume, or cerebral blood flow compared to controls^5,17,18^, others have observed increased GM volumes in specific brain regions such as the hippocampus and insula^19,20^. Additionally, white matter hyperintensities have been documented^21^. However, the collective implications of these findings and their relationship with the cognitive impact observed in PACS patients remain unclear, and as of now, there is no definitive consensus on the pathogenic mechanisms underlying PACS.

This study has two primary objectives. First, we aim to characterize the cognitive profile of patients presenting with cognitive complaints in PACS and track their recovery over a period of six months. Secondly, we seek to evaluate various markers of neuronal damage and inflammation through structural MRI and fluid markers to investigate cross-sectional and longitudinal associations with clinical and cognitive features. Our approach to studying PACS will integrate data from cognition, MRI scans, and fluid biomarkers, providing a comprehensive description of these participants and their evolution over time.

## Materials and methods

### Participants

We performed a prospective evaluation of patients referred to the Alzheimer’s disease and other Cognitive Disorders Unit of the Neurology Service at the Hospital Clínic de Barcelona, Barcelona, Spain. Participants were consecutively recruited between March 2021 and November 2021. Inclusion criteria were: (1) COVID-19 diagnosis, based on biological or clinical diagnosis (polymerase chain reaction, antigenic rapid detection test or microbiological test); (2) Cognitive symptoms reported by the participant or an observer (family member, co-worker, health professional); (3) presence of cognitive symptoms ≥ 8 weeks after COVID-19 symptoms onset; (4) fluent in Spanish; (5) at least 6 years of formal education; (6) age 35–65 years old (participants above the age of 65 years were not included in order to avoid a possible confusion factor with onset of neurodegenerative diseases symptoms). Exclusion criteria were: (1) Previous diagnosis of any neurological, psychiatric, or medical condition that could affect the baseline cognitive performance, including previous chronic fatigue syndrome diagnosis; (2) any condition that prevented the completion of the cognitive assessment and/or MRI scanning.

This study was performed according to the international consensus for research with human subjects (the updated version of Helsinki’s Statement, Fortaleza, 2013) and Spanish regulations. The Hospital Clínic de Barcelona Ethics Committee (HCB/2020/1483) approved the study, and all participants provided informed consent.

### Clinical and neuropsychological assessment

Participants were evaluated at baseline, 1 month, 3 months, and 6 months of follow-up. The time between the COVID-19 infection and the baseline visit was 10.4 (SD 3.9) months. Participants underwent general and neurological assessments and a comprehensive neuropsychological (NPS) battery administered by a trained neuropsychologist. The battery included estimated premorbid IQ (Spanish Word Accentuation Test)^22^, verbal memory tests: Free and Cued Selective Reminding Test (FCSRT)^23^; visual memory tests: Rey-Osterrieth Complex Figure Test (ROCFT)^24^ Recall; language tests: Boston Naming Test^25^, Vocabulary, semantic fluency^26^; visuospatial abilities: ROCFT Time; and attention and executive function tests: Trail Making Test (TMT) A and B^27^, Stroop Test^28^, Symbol Digits Modalities Test (SDMT)^29^, Digit Span Test^30^, Letter-Number sequencing^30^, Symbol Search^30^, and phonemic fluency^31^. Raw scores were transformed to scalar scores adjusted by age and years of education, with a normal distribution and a mean of 10. Abnormal cognitive performance was defined as having a scalar score lower than seven (< 7) in one or more cognitive subtests. This cutoff score of less than seven (< 7) was selected to be in line with what is considered clinically relevant, that is aligning with accepted practice in the clinical settings^32,33^. The participants also completed the Beck Depression Inventory (BDI)^34,35^, the Beck Anxiety Inventory (BAI)^36^, the Starkstein Apathy Scale (SAS)^37^, the Subjective Cognitive Decline Questionnaire (SCD-Q)^38^, the Multidimensional Fatigue Inventory (MFI-20)^39^ and the 36-Item *Short Form Health Survey (SF-36)*^40–42^. The NPS battery was identical in all the visits (baseline, 1 month, 3 months, and 6 months of follow-up).

### Neuroimaging studies

MRI scanning was performed at inclusion and the end of the study (6 months) using a 3T Prisma Siemens (Siemens Medical Systems, Germany) with the same MRI protocol. A high-resolution 3D structural dataset (T1-weighted, MP-RAGE, repetition time = 2.400 ms, echo time = 2.22 ms, 208 slices, field-of-view = 256 mm, 0.8 mm isotropic voxel) was acquired for everyone at each time. We used the processing stream available in FreeSurfer version 6.0 (http://surfer.nmr.mgh.harvard.edu.sire.ub.edu/) to perform cortical reconstruction and volumetric segmentation of the T1-weighted acquisitions. FreeSurfer allowed us to obtain cortical thickness (CTh) maps and segment the subcortical structures. For longitudinal data, we used the longitudinal stream in FreeSurfer. All FreeSurfer preprocessing steps are reported in detail elsewhere^43–45^. From the reconstructed data, we obtained global measures of mean CTh and grey matter (GM) volumes of the left and right hemispheres. In addition, we used the summary measures of mean CTh in 68 cortical parcellations and GM volumes of 14 subcortical structures, all derived from atlases available in FreeSurfer^46,47^. All images and individual segmentations underwent visual inspection and manual correction, if necessary, by one of the authors of this study (APM). This quality control process verified the accuracy of white matter and gray matter segmentation obtained with FreeSurfer. In cases where corrections were required, manual adjustments were made to the mask, followed by rerunning the FreeSurfer stream to ensure optimal segmentation.

### Biological measures

Blood samples were obtained at baseline (n = 49), at 1 month (n = 48), 3 months (n = 47), and 6 months (n = 46). An optional lumbar puncture to obtain cerebrospinal fluid (CSF) was offered to the participants at the basal visit (n = 12). Serum levels of neurofilament-light (NfL) and glial fibrillary acidic protein (GFAP) were determined by single molecule array technology (Neurology 2-Plex B Simoa, Quanterix®). A panel of cytokines, chemokines and other soluble mediators that included interferon (IFN)-α, β, and γ, interleukins (IL) IL-1β, IL-6, IL-8, IL-10, IL-17A, IL-18, tumor necrosis factor (TNF)-α2, IL-1 receptor antagonist (IL-1ra), Interferon-γ-Inducible Protein 10 (IP-10), granulocyte colony-stimulating factor (G-CSF), antigen CD25, chemokine ligand 1 (CX3CL1 or fractalkine), chemokine ligand 2 (CCL2), chemokine ligand 7 (CCL7) and ligand 9 (CXCL9) was analyzed in serum and CSF by a multiplexed bead based assay (Human Cyto Panel A, Merck, Germany) in a Luminex®100/200 platform. In addition, a tissue-based assay (TBA) consisting of an indirect immunohistochemistry (IIHC) with rat brain tissue and an indirect immunofluorescence assay (IIFA) with live neurons were performed to screen anti-neuronal immunoreactivity^48,49^.

The prospective study did not include cognitive normal non-PACS controls. As most of the biochemical measures evaluated here lack established cut-offs for normal values^50–52^, we included in the analysis 38 serum and 24 CSF samples from non-COVID-19 healthy controls from a previous study in acute COVID-19^14^. Normal results were defined as results within two standard deviations of the mean of the control group. We conducted biochemical analyses using identical kits and protocols for both healthy controls and PACS participants, at both baseline and follow-up assessments.

### Statistical analysis

Raw cognitive scores were converted to scalar scores (SS) according to age and number of formal education, with a normal distribution with a mean scalar score of 10. Abnormal cognitive performance was set at SS < 7, which is the cutoff point used in clinical practice. If one subtest or more showed abnormal scores, the evaluation was considered abnormal.

We first evaluated if cognitive test results differed in participants who scored within the normal range versus the pathological range on measures of anxiety, depression, apathy, fatigue, or quality of life scores. For that, we used previously described cut-offs: BDI ≥ 20 indicated moderate or severe depression, BAI ≥ 16 defined moderate or severe anxiety, SAS ≥ 14 was considered clinically significant apathy, SCD-Q ≥ 7 was considered pathological, MFI-20 cutoff of ≥ 60 was used for the description of a high-level versus low-level fatigue. SF36 subscales cutoff of ≥ 50 indicated normative scores; the reference population has a mean of 50 and a standard deviation of 10. We used permutation tests, adding age, sex, and years of education as covariates. Then, we studied the partial correlation between cognitive measures and physical and mental health scores with continuous variables and added age, sex, and years of education as covariables.

We also analyzed the neuropsychological results with a principal component analysis (PCA), a dimensionality reduction method. It was conducted to elucidate the key factors contributing to the highest variability in the dataset. Tests showing a higher prevalence of alterations among participants, such as verbal memory and attention/executive function tests from the NPS, mental health and subjective cognitive decline questionnaires (BDI, BAI, SAS, and SCD-Q), were included. By analyzing the first component and the individual contribution of each variable to it, we estimated which variables explained the highest variability in the data.

To assess the relationship between cognitive and mental health outcomes and MRI measurements, we conducted partial correlation analyses. These analyses focused on global and regional MRI metrics in relation to memory, executive function, anxiety, depression, fatigue, and subjective cognitive complaints (SCD). We included age, sex, and years of education as covariables. All analyses were corrected for multiple comparisons.

We measured the partial correlation of inflammatory soluble mediators, NfL, and GFAP levels with SCD, memory and executive function outcomes, anxiety, depression, fatigue, and global and regional MRI measures and added age and sex as covariables. All analyses were corrected for multiple comparisons using the Benjamini–Hochberg adjustment.

We performed longitudinal analyses including the baseline and the three follow-up visits with linear mixed-effects (LME) models to study changes between visits in cognitive measures, physical and mental health scores, global and regional MRI measures, and biochemical values for all the available data in each case. For cognitive tests, age, sex, and years of education were added as fixed effects. In the MRI and biochemical studies, age and sex were considered fixed effects.

Statistical analyses of the cross-sectional and longitudinal results were carried out in the language R version 4.2.1 (https://www.r-project.org). The R packages most notable for the analyses have been coin, corrplot, factoextra, ggplot2, ggpubr, ggseg, lme4, lmerTest and rstatix.

## Results

### Clinical data and neuropsychological characteristics

Fifty-three participants were assessed, 49 were included in the study and 46 completed the follow-up. 39 (80%) participants included were women, the mean age was 50.1 (SD 7.9, range 35–64), mean years of education (YOE) 14 (SD 3), with mean time from the onset of acute symptoms of 10.4 months (SD 3.9). 10 (20%) participants needed hospitalization; 7 (14%) received oxygen support, including 2 (4%) admitted into intensive care units. Participants presented with multiple symptoms other than cognitive complaints: 43 (88%) referred fatigue, 30 (61%) headaches, 31 (63%) dyspnea, 24 (49%) arthralgias, 21 (43%) myalgias, 19 (39%) bowel rhythm disturbances, 12 (24%) anosmia, 13 (27%) dysgeusia and 5 (10%) intermittent febrile. Twenty (41%) participants were on sick leave at inclusion.

**Figure 1:**
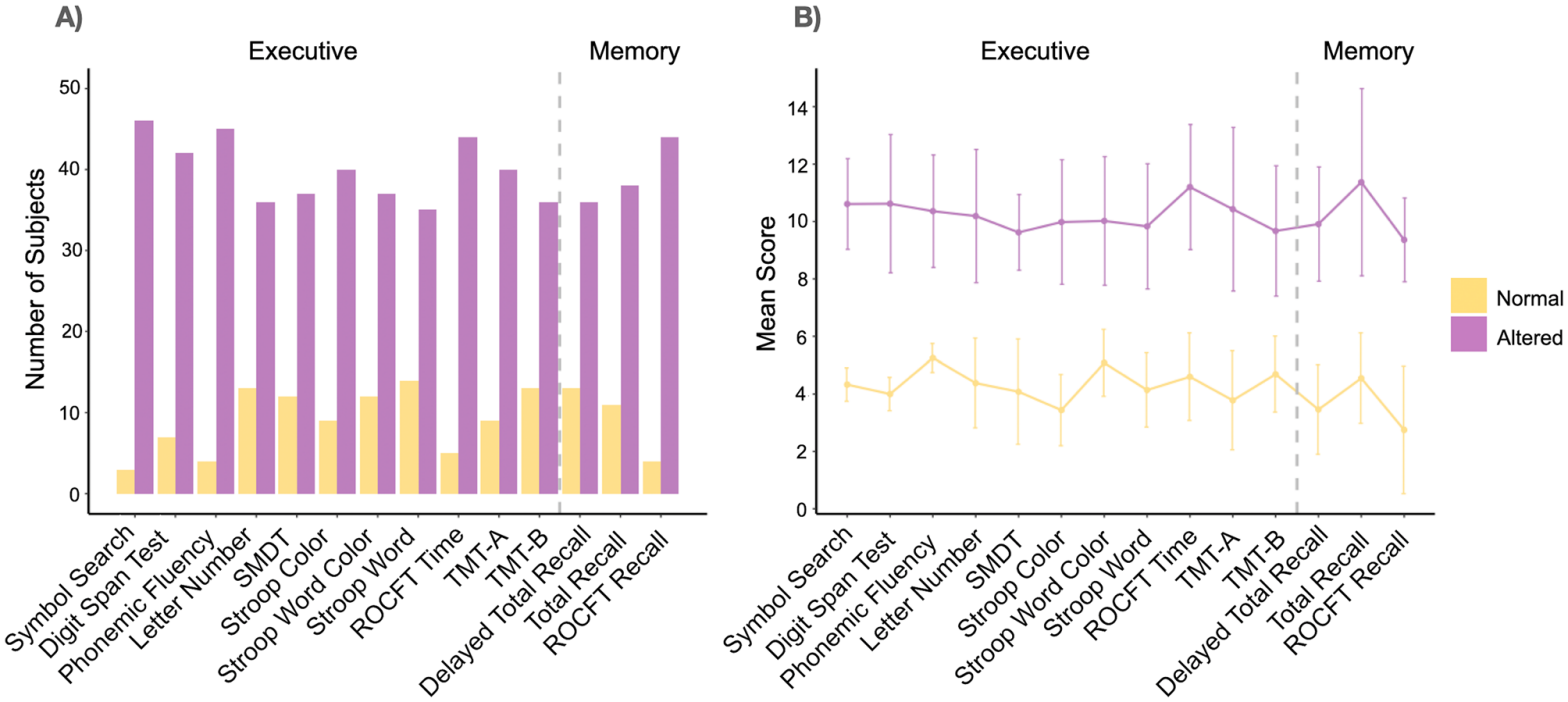
Neuropsychological test: memory and executive functions tests results. (**A**) Number of participants with altered/normal scores; (**B**) Mean scores of the neuropsychological tests. Scores are presented as scalar scores, adjusted by age and years of education, following a normal distribution with a mean of 10. Scores ≥ 7 are normal. The error bars represent the standard deviation.

The sample had high premorbid intelligence, estimated with the Word Accentuation Test. Twelve participants (24.5%) showed a normal cognitive evaluation defined by all test results within the limits of normality for age and years of education. The remaining participants (75.5%) presented abnormal scores in at least 1 test. Abnormal results were most frequently observed in executive functions and verbal memory (Table 1, Fig. 1). For this reason, further analysis with cognitive tests included only memory and executive functions tests.

**Table 1:** Neuropsychological test results.

Cognitive test	Baseline n = 49		+ 1 month n = 48		+ 3 months n = 47		+ 6 months n = 46	
	Mean score (SD)	n altered, %	Mean score (SD)	n altered, %	Mean score (SD)	n altered, %	Mean score (SD)	n altered, %
Memory								
FCSRT Free learning	8.6 (3.4)	13 (27%)	11.4 (3.3)	4 (8%)	12.4 (3.3)	4 (9%)	12.7 (3.7)	3 (7%)
FCSRT Total learning	9.8 (4.1)	11 (22%)	12.5 (4.7)	6 (12%)	13.1 (4.0)	2(4%)	14.2 (4.1)	4 (9%)
FCSRT delayed free recall	8.2 (3.4)	13 (27%)	9.7 (3.5)	9 (19%)	11.4 (3.2)	3 (6%)	11.3 (3.0)	2 (4%)
FCSRT delayed total recall	10.2 (4.9)	8 (16%)	13.5 (5.1)	4 (8%)	13.2 (5.2)	5 (11%)	12.9 (4.9)	4 (9%)
ROCFT recall	8.8 (2.4)	4 (8%)	10.3 (3.0)	2 (4%)	11.0 (2.8)	3 (6%)	10.9 (2.6)	0 (0%)
Language								
Boston naming test	12.2 (3.3)	2 (4%)	12.7 (3.5)	2 (4%)	12.7 (3.3)	1 (2%)	13.5 (3.6)	1 (2%)
Semantic fluency	9.8 (2.8)	5 (10%)	10.1 (3.1)	3 (6%)	10.3 (3.2)	4 (9%)	10.7 (2.8)	3 (7%)
Vocabulary	11.3(2.2)	2 (4%)	11.3 (1.9)	1 (2%)	11.5 (2.3)	1(2%)	11.8 (1.7)	1 (2%)
Visuoespatial abilities								
ROCFT copy	12.1 (3.8)	2 (4%)	10.7 (2.9)	1 (2%)	12.9 (3.5)	1 (2%)	12.6 (3.7)	1 (2%)
ROCFT time	10.5 (2.9)	5 (10%)	11.1 (3.3)	5 (10%)	11.9 (3.0)	2 (4%)	12.3 (3.2)	2 (4%)
Attention and executive function								
TMT-A	9.2 (3.7)	9 (18%)	9.6 (3.9)	7 (15%)	11.0 (3.8)	3 (6%)	11.3 (4.1)	5 (11%)
TMT-B	8.3 (3.0)	13 (27%)	9.0 (3.4)	9 (19%)	10.3 (3.6)	6 (13%)	9.6 (3.6)	8 (17%)
Phonemic fluency	9.9 (2.3)	4 (8%)	10.1 (2.4)	5 (10%)	10.5 (2.7)	4 (9%)	10.6 (2.6)	4 (9%)
Digit span—forward	9.7 (3.2)	7 (14%)	9.0 (3.0)	9 (19%)	9.5 (2.8)	6 (13%)	9.3 (3.1)	11 (24%)
Letter-number sequencing	8.7 (3.4)	13 (27%)	8.7 (3.1)	8 (17%)	8.4 (2.3)	8 (17%)	8.7 (2.7)	7 (15%)
SMDT	8.3 (2.8)	12 (24%)	8.9 (3.5)	10 (21%)	9.8 (3.3)	6 (13%)	9.8 (3.3)	6 (13%)
Symbol search	10.2 (2.2)	3 (6%)	10.2 (2.4)	2 (4%)	10.9 (2.3)	1(2%)	10.9 (2.1)	1 (2%)
Stroop word	8.2 (3.3)	14 (29%)	8.2 (2.8)	14 (29%)	8.7 (2.6)	10 (21%)	8.5 (2.7)	10 (22%)
Stroop color	8.8 (3.3)	9 (18%)	8.6 (2.9)	10 (21%)	8.9 (2.8)	8 (17%)	8.3 (3.1)	12 (26%)
Stroop word-color	8.8 (2.9)	12 (24%)	8.8 (3.1)	11 (23%)	9.0 (3.1)	9 (19%)	9.0 (2.6)	9 (20%)

*FCSRT* Free and Cued Selective Reminding Test, *ROCFT* Rey-Osterrieth Complex Figure Test, *SD* standard deviation, *SMDT* Symbol Digits Modalities Test, *TMT* Trail Making Test, *VOSP* Visual Object and Space Perception Battery. Scores are presented as scalar scores, adjusted by age and years of education, following a normal distribution with a mean of 10. Scores ≥ 7 are considered normal and < 7 are considered altered in clinical practice. The right columns show the mean scalar scores and standard deviations. The left columns show both the absolute number and the percentage of participants with altered scores.

The mental health assessments conducted in our study revealed widespread and significant impairment across various domains, including depression, anxiety, apathy, fatigue, and quality of life.

Out of the total participants, nine (19%) exhibited characterized to severe depression based on questionnaire results (BDI) (Table 2). 26 participants (57%) exhibited moderate to severe anxiety based on questionnaire results (BAI). 29 participants (64%) exhibited clinically significant apathy (SAS). Subjective cognitive decline reports of participants (MyCog) were altered, indicating worsening in 48 out of 49 (98%) cases, while reports of proxies (TheirCog) showed alterations in 33 out of 44 (75%) cases (Table 1). The fatigue scale MFI-20 showed a mean of 57.7/100, and 35.4% of the sample fell within the severe fatigue group (Table 3).

**Table 2:** Subjective cognitive decline and psychiatric symptoms questionnaires.

	Baseline	Follow-up
Beck Anxiety Inventory (0 to 63)	n = 46	n = 39
Minimal (0 to 7)	4 (9%)	8 (21%)
Mild (8 to 15)	16 (35%)	11 (28%)
Moderate (16 to 25)	12 (26%)	10 (26%)
Severe (26 to 63)	14 (30%)	10 (26%)
Beck Depression Inventory (0 to 40)	n = 46	n = 40
Minimal (0 to 13)	22 (48%)	20 (50%)
Mild depressive symptoms (14 to 18)	15 (33%)	8 (20%)
Moderate clinical depression (19 to 27)	6 (13%)	8 (20%)
Severe depression (28 to 63)	3 (6%)	4 (10%)
SCD-Q MyCog (0 to 24)	n = 46	n = 41
Normal (0 to 6)	1 (2%)	4 (10%)
Pathological (7 to 24)	45 (98%)	37 (90%)
SCD-Q TheirCog (0 to 24)	n = 45	n = 41
Normal (0 to 6)	12 (27%)	11 (27%)
Pathological (7 to 24)	33 (73%)	30 (74%)

Changes over time: Despite minor fluctuations observed in score distributions over time, longitudinal analysis using an LME model revealed no significant changes in anxiety, depression, or subjective cognitive decline (SCD) scores at the 6-month follow-up SCD-Q, subjective cognitive decline questionnaire.

**Table 3:** Multidimensional Fatigue Inventory (MFI-20).

Multidimensional fatigue inventory (MFI-20)	Baseline mean score (SD)	Follow-up (6 months post-baseline) mean score (SD)
Total score (20 to 100)	57.7 (5.1)	61.3 (6.1)
Mental fatigue (4 to 20)	10.2 (2.6)	10.8 (3.0)
General fatigue (4 to 20)	11.3 (1.9)	12.2 (2.1)
Physical fatigue (4 to 20)	12.3 (2.0)	12.8 (2.1)
Reduction of activity (4 to 20)	12.9 (2.1)	12.9 (1.6)
Reduction of motivation (4 to 20)	11.7 (2.9)	13.0 (2.3)

SD, standard deviation. The MFI-20 Total Score ranges from 20 (no fatigue) to 100 (maximum fatigue). Subscales Scores range from 4 (no fatigue) to 20 (maximum fatigue). Higher scores indicate more fatigue. The total score is obtained from the sum of the subscale scores.

Moderate depression, characterized by notable distress and daily functional impairment, is associated with the presence of persistent symptoms like sadness, feelings of worthlessness, or guilt. In contrast, severe depression presents intense symptoms that profoundly disrupt daily life. Similarly, moderate anxiety is characterized by noticeable distress and impairment in daily functioning due to excessive worry and physical symptoms, whereas severe anxiety entails intense and debilitating symptoms, often including panic attacks and avoidance behaviors. Clinically significant apathy is defined as a pronounced and impairing lack of motivation and interest that significantly impacts daily functioning and quality of life.

The MyCog and TheirCog questionnaires assess subjective cognitive decline, with higher scores indicating a greater likelihood of cognitive impairment. In our study, the mean total score of 57.7 on the MFI-20 closely approaches the threshold of 60, which is recognized in some studies as indicative of severe fatigue, indicating that our study population is experiencing a significant level of fatigue.

The SF-36 evaluates health-related quality of life across eight domains, measuring scores from 0 (indicating the worst possible health state) to 100 (the best possible health state). For normative data, we refer to the averages within adults of working age (18–64 years), categorized by age and sex. Typically, mean scores are higher in men and tend to decrease with age. While the SF-36 does not have a universally agreed-upon cutoff score, a threshold of 50 is often used to identify impaired health-related quality of life. Given that our study population comprises 80% women with a mean age of 50.1 years, we have selected the mean scores of women aged 45–54 as our reference point, to contextualize our findings more effectively. In comparison to these normative values, participants experiencing PACS reported significantly lower scores in all eight domains of the SF-36, indicating a widespread impairment in health-related quality of life. Specifically, the mean scores for our participants versus controls in key domains were as follows: Physical Functioning 64.3 vs. 84.4, General Health 51.8 vs. 73.1, Role Limitations due to Physical Health 33.9 vs. 82.4, Pain 47.3 vs. 77.4, Emotional Well-being 56.2 vs. 80.8, Social Functioning 55.8 vs. 87.0, Role Limitations due to Emotional Problems 40.9 vs. 80.8, and Energy/Fatigue 29.8 vs. 59.4 (Table 4). These disparities underscore a significant decrease in quality of life among PACS sufferers across all measured domains.

**Table 4:** A 36-item short form survey instrument assessing health-related quality of life.

Subscales	Baseline		Follow-up	
	Mean score, SD	n altered, %	Mean score, SD	n altered, %
Physical functioning	64.3 (23.3)	9 (20.0%)	65.5 (27.3)	13 (32.5%)
General health	51.8 (17.4)	23 (51.1%)	48.9 (23.5)	23 (57.5%)
Role limitations due to physical health	33.9 (44.0)	29 (64.4%)	31.2 (41.1)	26 (65.05)
Pain	47.3 (28.2)	26 (57.8%)	46.1 (27.4)	23 (57.5%)
Emotional well-being	56.2 (18.0)	16 (35.6%)	49.9 (19.7)	21 (52,5%)
Social functioning	55.8 (26.4)	13 (28.9%)	52.3 (26.3)	17 (42.5%)
Role limitations due to emotional problems	40.9 (44.3)	27 (61.4%)	44.9 (44.4)	22 (55.0%)
Energy/fatigue	29.8 (18.6)	36 (80.0%)	28.4 (18.8)	34 (85.0%)

*SD* standard deviation. Scores range from 0 to 100. Although there is no universally established cutoff score for the SF-36, a cutoff of 50 is sometimes utilized as a threshold for identifying impaired health-related quality of life. Compared to normative data, participants with PACS demonstrated lower scores across all SF-36 domains. Results are presented as mean score (standard deviation), number and percentage of participants with score < 50.

We next sought to stratify participants by levels of anxiety, depression, apathy, fatigue, or quality of life scores according to their questionnaire scores. Participants displaying moderate or severe anxiety showed lower results in the ROCFT Recall subtest (adjusted p-value = 0.0014). No significant differences were observed in cognitive tests between participants with normal and abnormal values of the other stratification categories.

**Figure 2:**
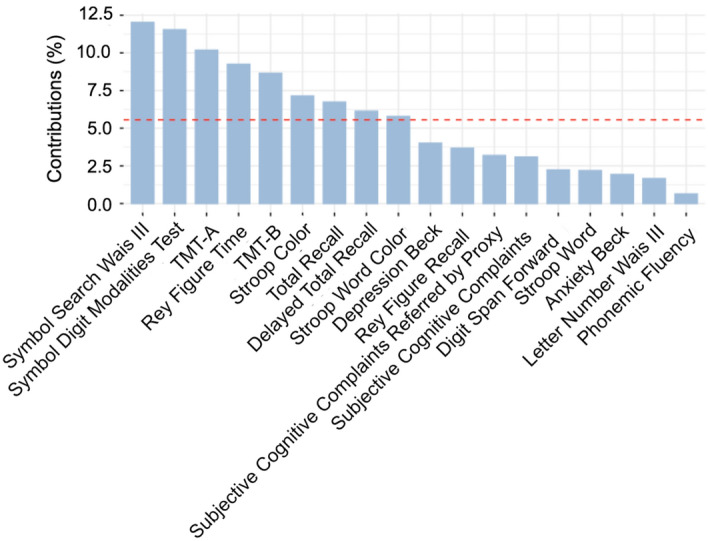
Principal component analysis results of neuropsychological studies. It shows the variables with the most contributions to the first component. The dashed red indicates the average contribution. A variable with a contribution greater than this limit is important to the principal component.

In our study, a PCA analysis demonstrated that executive function scores emerged as the primary determinants of variability within the PACS sample population's cognitive and mental health outcomes (Fig. 2). Notably, the three cognitive tests most influential in explaining the variability of the data were Symbol Search, SDMT, and TMT-A.

At 6 months follow-up using an LME model, cognitive test scores generally remained stable compared to baseline, except for the FCRST Delayed Free Recall test, which showed improvement. Specifically, the mean score increased from 10.0 at baseline to 12.4 after 6 months (p-value = 0.0040). Additionally, the proportion of participants with a normal NPS increased at follow-up (50%) compared to baseline (24%) (p-value = 0.012). However, no significant longitudinal differences were observed in depression, anxiety, apathy, fatigue, or SCD scores at 6 months.

### Correlations between neuroimaging and cognitive data

**Figure 3:**
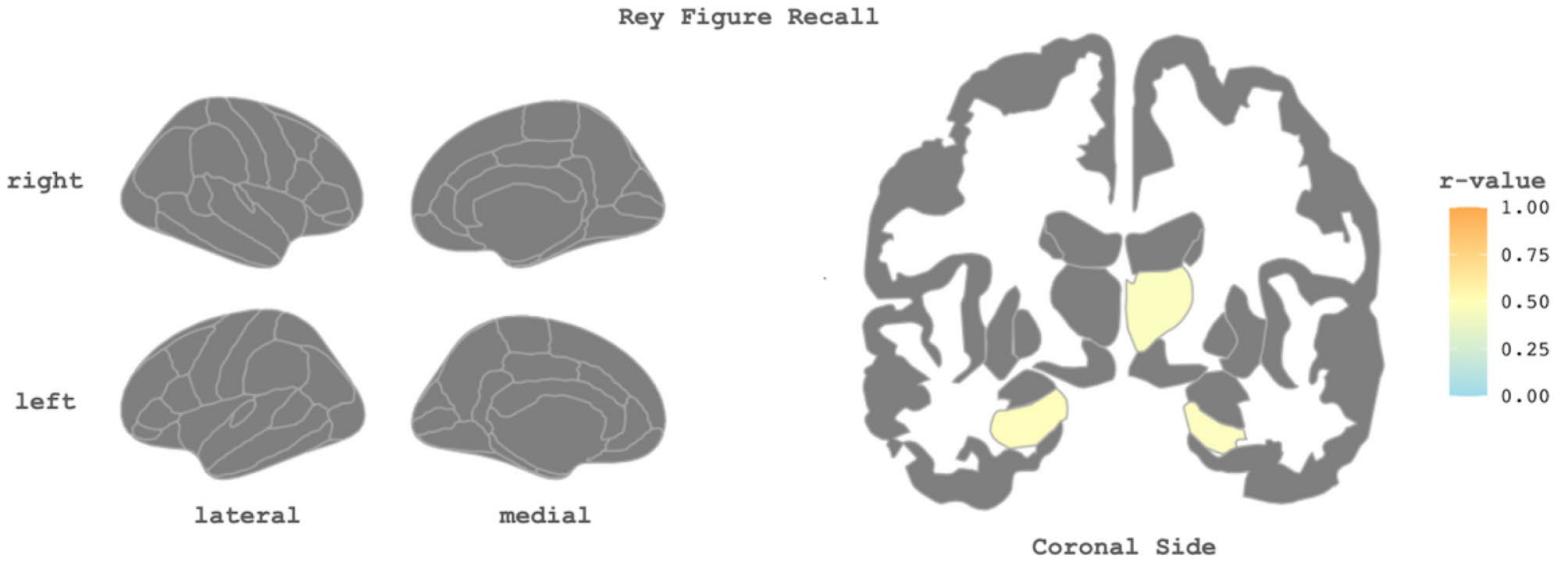
The brain plots display significant correlations between cortical thickness measures, gray matter volumes, and the Rey-Osterrieth Complex Figure Test (ROCFT) Recall at baseline. Significant correlations, corrected for multiple comparisons (p < 0.05), are highlighted, with the r-value indicated by the color bar, while the gray color indicates non-significant correlations. The left plot illustrates correlations between ROCFT Recall and cortical thickness measures. The right plot depicts correlations between gray matter volumes and ROCFT Recall.

To identify whether neuropsychological tests correlate with the neuroimaging data, partial correlations were performed at baseline. ROCFT Recall scores showed a moderate correlation with total GM volume (r = 0.46, adjusted p-value = 0.046), subcortical GM volume (r = 0.52, adjusted p-value = 0.024), and left cerebral white matter (WM) (r = 0.47, adjusted p-value = 0.046). At the regional level, the ROCFT Recall scores also showed a significant positive correlation with GM volume in left hippocampus GM (r = 0.51, adjusted p-value = 0.042), right hippocampus GM (r = 0.49, adjusted p-value = 0.042), and right thalamus GM (r = 0.48, adjusted p-value = 0.042, Fig. 3). Other cognitive tests and clinical outcomes (SCD, anxiety, depression questionnaires) were not significantly associated with global or regional structural MRI indexes after correction for age, sex, years of education, and multiple comparisons.

**Figure 4:**
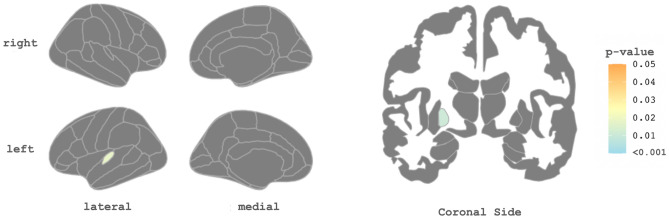
Longitudinal differences in regional MRI indexes (baseline vs 6-month follow-up). Significant correlations, corrected for multiple comparisons (p < 0.05), are highlighted, the significant regions of the liner fixed effects models indicated by the color bar. While the gray color indicates non-significant regions, The left plot illustrates cortical thickness measures and the right plot depicts gray matter.

When probing for longitudinal changes with an LME model, we did not identify significance in global structural MRI measures at 6 months in PACS participants compared with baseline. At the regional level, we found GM loss at 6 months in the GM volume of left pallidum (p-value = 0.0098) and left transverse temporal thickness (p-value = 0.018) (Fig. 4).

### Biochemical analyses

Finally, we assessed group serum levels of NfL, GFAP, and cytokines, as well as CSF levels of cytokines. We also sought to assess whether serum or CSF samples contained anti-neuronal immunoreactivity. We compared these biomarkers between PACS patients and controls. The results showed no significant differences in serum NfL and GFAP levels between the groups. However, several cytokines differed significantly: in serum, G-CSF, IP-10, MIG, and MCP1 were notably lower in PACS, while in CSF, IL-18 and IL-17a were higher, and IL-10, IL1AR, IL-8, IP-10, and MCP1 were lower in PACS patients. However, the magnitude of the differences was small, and all values were within the limits of normality (Supplementary Material). All the serum and CSF samples were negative for anti-neuronal immunoreactivity. Our longitudinal analysis using a LME model revealed no significant changes in cytokines, NfL, or GFAP levels in PACS participants over time, except for a significant decrease in IL-17a at the 6-month mark, which still remained within normal ranges. We found a moderate positive correlation between GFAP levels and the Stroop Word test scores (r = 0.48, adjusted p-value = 0.018) at baseline. Global and regional MRI indexes and cognitive scores did not significantly correlate with serum or CSF cytokine or NfL levels after adjusting for multiple comparisons.

## Discussion

In the present study, we conducted a comprehensive cross-sectional and longitudinal assessment of individuals with PACS regarding their cognition, mental health status, neuroimaging, and fluid biomarker profile. This approach offers a broad view of PACS patients, which is particularly valuable considering the limited previous research in this area. We evaluated PACS patients with subjective cognitive complaints and their evolution over a 6-month period. Our findings revealed cognitive impairment affecting executive function in more than two-thirds of participants and verbal memory in over one-third. Additionally, prevalent mental health issues included apathy (64%), moderate-severe anxiety (57%), and severe fatigue (35%). Visual memory impairment correlated with total gray matter and subcortical gray matter volume, as well as regional GM reductions in the hippocampus and thalamus. Notably, markers of neuronal damage and inflammation were within normal limits. Importantly, overall health and cognitive evaluations showed no significant change over time. Furthermore, altered executive function and verbal memory, common in PACS, persisted in most subjects without any link to alterations in their biomarker and imaging profiles.

Our study stands out for three main reasons: First, it assesses cognitive deficits through comprehensive neuropsychological evaluations. Whereas the literature on PACS is replete with studies on cognition using screening tools like the Mini-Mental State Examination or the Montreal Cognitive Assessment^53,54^, our research incorporates detailed neuropsychological evaluations conducted by an experienced neuropsychologist. Second, our study is distinguished by its longitudinal design. Beyond describing the alterations in patients suffering from PACS, we repeated the same analyses six months later to assess their progression and track the evolution of these key health indicators over time. Third, our study is notable for the breadth of areas evaluated: it meticulously examines cognition, mental health, brain structure, and markers of inflammation and neuronal damage concurrently, tracking their longitudinal evolution.

Cognitive evaluations in PACS showed that attention-executive and verbal memory were the most affected domains (Fig. 1), which has been described in previous published works; however, the pattern of alterations was broader and more heterogeneous between patients^4–7,52,53^. The sample had a high premorbid intelligence and would not be expected to perform below average on cognitive testing. Despite their cognitive reserve, known as a protective mechanism against neurological impairments, these individuals nonetheless experience cognitive deficits. This would likely lead to difficulties in effectively managing daily work and life responsibilities, adversely affecting their quality of life. At a 6-month follow-up, we determined that only the FCSRT Delayed Free Recall of verbal memory scores improved significantly from baseline, using LME models. Nevertheless, if we consider the percentage of normal evaluations (defined as the proportion of tests within clinical limits of normality), there was a significant improvement with time. Considering that most participants with abnormal results were close to the threshold for normal performance, even a slight improvement in these tests could lead to reaching normal threshold values. The improvement in verbal memory and the achievement of normality in the neuropsychological tests indicate a positive trajectory toward normal cognitive functioning. Conversely, high levels of anxiety, apathy, and fatigue present at the beginning of the study remained unchanged. The slight improvements observed in cognition did not strongly affect participants' clinical outcomes or quality of life. It is plausible that the persistence of executive function deficits, ongoing psychological symptoms, and chronic fatigue significantly influenced the overall lack of enhancement in participants' well-being.

Our results are in line with previous published works showing both improvements and persisting cognitive deficits in PACS^55,56^. Previous research has also documented a decline in executive functions among participants who initially presented with severe cognitive impairment^57^. The repeated administration of a cognitive test four times over a six-month period in our study raises concerns about the potential influence of a learning effect on the results. To mitigate the learning effect, participants received standardized instructions and practice trials during the baseline assessment to familiarize themselves with the cognitive test procedures, potentially minimizing the influence of initial unfamiliarity or anxiety on test performance. In future research, it is advisable to schedule evaluations at more significant intervals to allow for a more comprehensive study of PACS.

Participants also reported depressive symptoms, anxiety, apathy, fatigue, and low scores in general health. These symptoms did not improve during this 6-month study (Table 2). Given that our analysis demonstrated a significant relationship between one memory test and stratification in anxiety scores, we believe that the coexistence of cognitive and mental health symptoms could not be interpreted as causality. Additionally, the modest sample size in our study may have limited our ability to detect subtle differences in other categories. Recent literature has reported mixed findings regarding the associations between psychiatric comorbidities and cognitive impairment in individuals with PACS. For instance, one recent study identified a significant association between depression symptom severity and cognitive impairment severity among PACS patients^58^, while findings for post-traumatic stress disorder and anxiety were inconclusive. Conversely, another study found no association between depression, anxiety, total general health status, fatigue, and cognitive profiles^59^.

In our study of PACS participants, we observed a complex interplay between cognitive and mental health symptoms, with prevalent cognitive impairment alongside high levels of anxiety, apathy, and fatigue. This underscores the intertwined nature of cognitive and mental health domains in PACS, where cognitive deficits may coexist with psychiatric symptoms. We next sought to stratify participants by levels of anxiety, depression, apathy, fatigue, or quality of life scores according to their questionnaire scores. Participants displaying moderate or severe anxiety showed lower results in the ROCFT Recall subtest (adjusted p-value = 0.0014). No significant differences were observed in cognitive tests between participants with normal and abnormal values of the other stratification categories. No associations were detected between longitudinal changes in cognitive and mental health measures. Conversely, a previously published work found that changes in executive functions were significantly associated with changes in depressive symptoms^57^.

While we can hypothesize that mental health issues may impede cognitive symptom improvement, it is worth noting that these mental health issues could be a consequence of the cognitive impairment as described elsewhere^60^. Understanding this relationship is crucial for informing treatment approaches; interventions targeting cognitive rehabilitation should consider the impact of comorbid psychiatric symptoms. Integrated interventions addressing both cognitive and psychiatric symptoms concurrently may optimize patient outcomes in PACS. Furthermore, both types of symptoms may be influenced by fatigue, which was nearly universal and severe in 35% of participants.

Fatigue has been implicated in various aspects of cognitive function, including attention, processing speed, and executive function, and has been associated with cognitive impairment in other medical conditions such as fibromyalgia and chronic fatigue syndrome. Moreover, fatigue often coexists with psychiatric symptoms such as anxiety and depression, contributing to the complex interplay between cognitive and mental health domains. In a recent study by Delgado-Alonso et al.^61^ investigated the relationship between subjective cognitive complaints, cognitive function, fatigue, and neuropsychiatric symptoms using various analytical methods. The study found that fatigue played a central role as the main mediator between objective and subjective cognition, while the impact of depression was indirect and mediated through fatigue. The lack of symptom improvement in PACS during the study suggests complex underlying factors. Possible reasons include the chronic nature of PACS, ongoing inflammation, and the interplay between cognitive and mental health symptoms. This highlights the need for personalized, multidisciplinary treatment approaches. Strategies may include pharmacological interventions, cognitive rehabilitation, psychotherapy, and lifestyle modifications.

Our study revealed a specific connection between cognitive deficits and brain changes in individuals with PACS. The ROCFT Recall test, a measure of memory and visual-spatial abilities, was the only cognitive test that showed abnormalities. These abnormalities were connected to both overall and specific areas of brain volume loss, specifically in the GM and WM globally, and in particular regions like the hippocampus and thalamus. This finding is significant because it identifies a direct relationship between certain cognitive deficits and changes in brain structure among individuals with PACS. The fact that these links were observed globally in GM and WM volumes, as well as in specific regions critical for memory and cognition (the hippocampus and thalamus), underscores the potential impact of COVID-19 on brain health. However, the fact that these associations were limited to certain brain regions and were only detected with the ROCFT Recall test suggests that the structural brain changes in PACS might be more nuanced than previously understood. While other studies, such as the one by Díez-Ciranda et al.^5^, have also found connections between cognitive deficits and MRI results, the limited scope of these associations in our study points to a potential gap in the literature. Specifically, it raises questions about the extent and significance of brain structural changes in PACS. Although it was beyond the scope of our study, it's noteworthy that some researchers have investigated the utility of functional neuroimaging to deepen our understanding of PACS pathophysiology. Bungenberg et al., in a cross-sectional study, used resting-state functional MRI (fMRI) to examine participants with PACS. They discovered changes in several brain regions—including the brainstem, olfactory cortex, cingulate cortex, thalamus, and cerebellum—on average seven months after SARS-CoV-2 infection. These alterations were associated with the severity of fatigue and cognitive functioning^54^. While structural MRI delineates the brain anatomy, fMRI sheds light on the brain’s dynamic functions. By revealing changes in brain activity and connectivity, fMRI could reveal underlying neural mechanisms of PACS that are not apparent in structural changes alone.

Our next approach in this study was to correlate clinical and neuroimaging features of this PACS cohort longitudinally. While a previous study has included both cognitive and neuroimaging assessment of PACS^62^, to our knowledge, this is the first study to include longitudinal analysis of both cognitive and neuroimaging tests. We found significant positive correlations between both global and focal measures of brain volume/thickness and visual memory scores, but not with other cognitive tests. This correlation indicated that worse visual memory was associated with lower total and subcortical GM volume together with left cerebral WM volume. Furthermore, subcortical GM volumes, especially the hippocampus and thalamus, significantly corresponded with worse visual memory performance. Previous studies also explored the association between GM volume and cognitive symptoms; it has been reported that worse memory and visuospatial test performance is associated with a loss of GM volume^5,20^. In line with previous studies, our longitudinal analyses revealed no evidence of volume gain in a 6-month period, nor did we find evidence of progressive volume loss broadly. However, we did observe significant gray matter loss in the left pallidum and left transverse cortical thickness. Despite these findings, we do not believe that they hold clinical significance. The observed changes in the left pallidum and left transverse cortical thickness were not associated with any clinical symptoms or functional impairments in our study population. Therefore, we do not interpret these findings as clinically meaningful^54^.

The majority of previous studies^13,14,16,63–66^, have reported high levels of plasma and/or CSF cytokines, NfL and GFAP in the acute or subacute phase of COVID-19 infection that normalize at follow-up, albeit using differing follow-up intervals^64,67,68^. Some of these studies related these biochemical changes with the severity of the infection or the gravity of neurological symptoms; however, there is no consensus on how fluid biomarkers relate to acute COVID-19 symptom severity, PASC symptoms, or PASC progression/resolution. In our study, the levels of plasma and CSF cytokines, NfL and GFAP were within pre-specified normal limits. Similar results were observed by Boesl et al.^69^, they found that NfL levels were normal in participants with self-reported cognitive complaints, and GFAP was altered in only 4%. They compared participants with subjective cognitive decline, single domain or multi-domain impairment and found no association between persistent neuronal or astrocytic damage and cognitive impairment. We observed slight differences in some cytokine levels between PACS and control participants, with variations of small magnitude. Furthermore, cytokine levels were either elevated or reduced compared to controls. Given the proximity of all values, even minimal differences in a subset of measurements could potentially lead to clinically significant results. Despite achieving statistical significance, we find this difficult to interpret and potentially inconclusive, and in our opinion, without clinical significance. However, it is worth mentioning that other studies in neurocognitive disorders show relationships between select cytokines with measures of cognitive function, and this warrants further examination. We did not observe significant differences in either GFAP or NfL levels between PACS participants relative to controls. Previous studies^14–16,70,71^ have inconsistent results regarding the association of fluid biomarkers with the severity of infection or neurological symptoms. This variability in findings from past studies may arise from methodological differences, diverse patient populations, and the dynamic nature of the post-acute phase of COVID-19. All the samples were negative for antineuronal antibodies. The absence of antineuronal antibodies in all samples holds clinical significance, suggesting that autoimmunity involving these specific antibodies may not be a predominant factor in the pathophysiology of PACS. This finding implies that cognitive impairment and neurological symptoms observed in PACS may be driven by mechanisms other than direct autoimmune responses targeting neurons.

We next sought to clarify whether these biochemical markers related to neuropsychological test results in PACS patients, as previous studies have inconsistent results regarding the association of inflammatory marker levels and neuropsychological tests. Results have ranged from no association^72^ to an association between cytokine levels and fatigue or executive functions (Stroop Color Word test)^73^, or TNF-α levels and memory^74^. In our research, we discovered a surprising positive correlation between higher GFAP levels and enhanced Stroop Word test performance. Despite observing impairment in Stroop Word test results among participants, GFAP levels stayed within normal ranges, suggesting these levels might not substantially affect cognitive performance or act as a cognitive function marker. Contrary to the expected negative correlation between GFAP and cognitive testing, stemming from inflammation's assumed detrimental effects on cognition, our findings suggest otherwise. This could indicate a compensatory or specific role of glial cell activation in supporting cognitive functions, or potentially represent a Type I error. This intriguing result encourages further investigation to confirm these findings and uncover the mechanisms involved. No association was observed between cytokines, NfL, or GFAP levels and global or regional MRI measures after adjusting for multiple comparisons. Finally, we found that patients’ serum or CSF samples did not immunoreact with brain tissue or live neurons, suggesting that brain autoantibodies are not involved in PACS symptoms. While our study did not reveal any significant abnormalities in markers of neuronal damage, inflammation, or neuroimaging among individuals experiencing cognitive manifestations following COVID-19 infection, several potential pathophysiological mechanisms warrant consideration. It is plausible that subtle, yet to be identified systemic or central dysregulated immune responses or diffuse microvascular or barrier changes could contribute to cognitive manifestations. We might also consider the central role of fatigue in cognitive manifestations. Future research exploring these mechanisms in depth is crucial for a comprehensive understanding of the neurological sequelae of COVID-19 infection.

An interesting finding elucidated by this work is the breakdown of PACS amongst sex. Whereas COVID-19 infects women and men equally, related publications indicate that there is a higher prevalence of females with PACS, with percentages ranging from 63 to 74%^13,16,75^, in line with these observations, 79% of participants in this study were women. Interestingly, in a study including 377 patients with COVID-19 infection, the female sex was independently associated with PACS within the multivariable analysis^75^. The higher prevalence of PACS in females suggests multifaceted influences across biological, psychological, and social dimensions. Hormonal differences may affect immune responses and neuroinflammation, contributing to gender-based variations in susceptibility and outcomes. Psychologically, gender-specific stressors and coping mechanisms could impact symptom manifestation, requiring exploration of psychosocial aspects in the post-acute phase. Social disparities, including healthcare-seeking behavior and societal expectations, may further influence the identification and reporting of PACS symptoms. Acknowledging these complexities highlights the importance of customized research and treatment approaches for effectively addressing PACS in females.

A significant limitation of our study is the small sample size, which included only 49 participants at baseline and 46 at the 6-month follow-up visit. This limitation is particularly pronounced concerning CSF samples. The lumbar puncture procedure was designated as optional. Consequently, CSF samples were obtained from only 12 participants. As a result, the interpretation of our findings must be approached cautiously; a larger number of CSF samples would have provided a more robust basis for identifying differences compared to controls, if any. The statistical analyses have been adapted to the reduced data. Thus, it could be generalized our results. However, the unicentric nature of the study, even if limited in the sample size, also provided homogeneity to the data acquisition. Secondly, the present study neither has healthy participant controls nor participants with COVID-19 infection without cognitive complaints for neuropsychological or neuroimaging analyses. This was due to the review of the local Ethics Committee, which considered the inclusion of controls as too high of a demand. This study may face referral bias, as participants were referred by healthcare providers, potentially overrepresenting severe cases. Additionally, considering the heightened fear and anxiety surrounding COVID-19, some participants actively sought assistance and self-referred due to concerns about cognitive symptoms related to the virus. Further research should consider a more diverse and randomized sample to mitigate potential biases in interpreting the severity and prevalence of cognitive symptoms in PACS. Another limitation of the study lies in the absence of baseline cognitive assessments prior to COVID-19 infection, which could have offered valuable insights into pre- and post-infection cognitive changes. However, participants with previous cognitive impairment were excluded. Finally, we believe the current duration of this study was limited and that including a longer endpoint with greater distance between measurement intervals may be more suitable for studying PACS cognitive symptoms. However, the study was designed during the last quarter of 2020, even before the formal definition of PACS, and most studies then were designed with short follow-up periods^20,64^.

In conclusion, our study showed cognitive impairment, mainly affecting attention/executive and verbal memory functions lasting for at least 6 months in individuals with PACS. Cognitive impairment was accompanied by depressive symptoms, apathy, anxiety, fatigue, and low health status. These findings (except for visual memory loss) were not associated with brain structural abnormalities, elevated cytokines, markers of neuronal damage, or neuronal antibodies. Given these findings, a tailored and multidisciplinary approach involving cognitive and mental health interventions is recommended for patient care. Future research is essential for understanding the enduring cognitive trajectory of PACS and the associated biological mechanisms. Longitudinal studies of extended duration will provide insights into the long-term cognitive impact. Addressing the gaps identified by our study's limitations, ongoing research endeavors are crucial to guide clinical interventions and enhance the overall management of PACS.

### Supplementary Information

Supplementary Figures.

## Data Availability

The datasets used and analyzed during the current study are available from the corresponding author upon reasonable request.
