# Supplementary material for: Cognitive profile, neuroimaging and fluid biomarkers in post-acute COVID-19 syndrome
Source: Sci Rep. 2024 Jun 5;14:12927. doi: 10.1038/s41598-024-63071-2 (PMC11153491; doi:10.1038/s41598-024-63071-2)
Supplement: Supplementary file 1 — Supplementary Figures. [file 41598_2024_63071_MOESM1_ESM.docx]

**Supplementary Material: Cognitive profile, neuroimaging and fluid biomarkers in post-acute COVID-19 syndrome**

**Núria Guillén^*^, Agnès Pérez-Millan^*^, Neus Falgàs, Gema M Lledó-Ibáñez, Lorena Rami, Jordi Sarto, Maria A Botí, Cristina Arnaldos-Pérez, Raquel Ruiz-García, Laura Naranjo, Bárbara Segura, Mircea Balasa, Roser Sala-Llonch , Albert Lladó, Sarah M. Gray, Jason K. Johannesen, Madeline M. Pantoni, Grant A. Rutledge, Ruta Sawant, Yi Wang, Luke S. Watson, Josep Dalmau, Raquel Sanchez-Valle**

* These authors contributed equally

## Cytokines Analysis


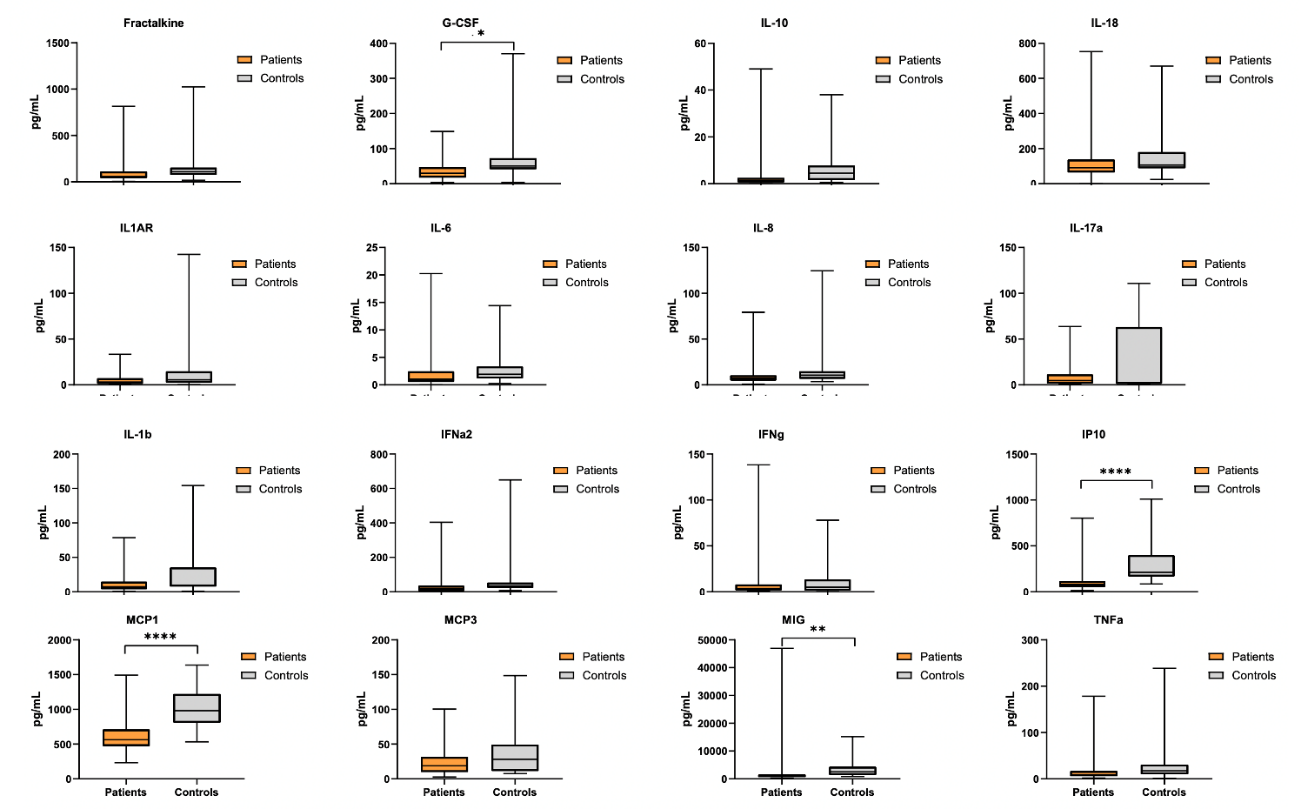


Figure 1: Boxplot of the serum cytokines levels. In the analysis, we had 49 patients (Post-acute COVID-19 syndrome) and 38 controls (healthy controls). Indicates *p < 0.05, **p < 0.01, ***p < 0.001, and ****p < 0.0001


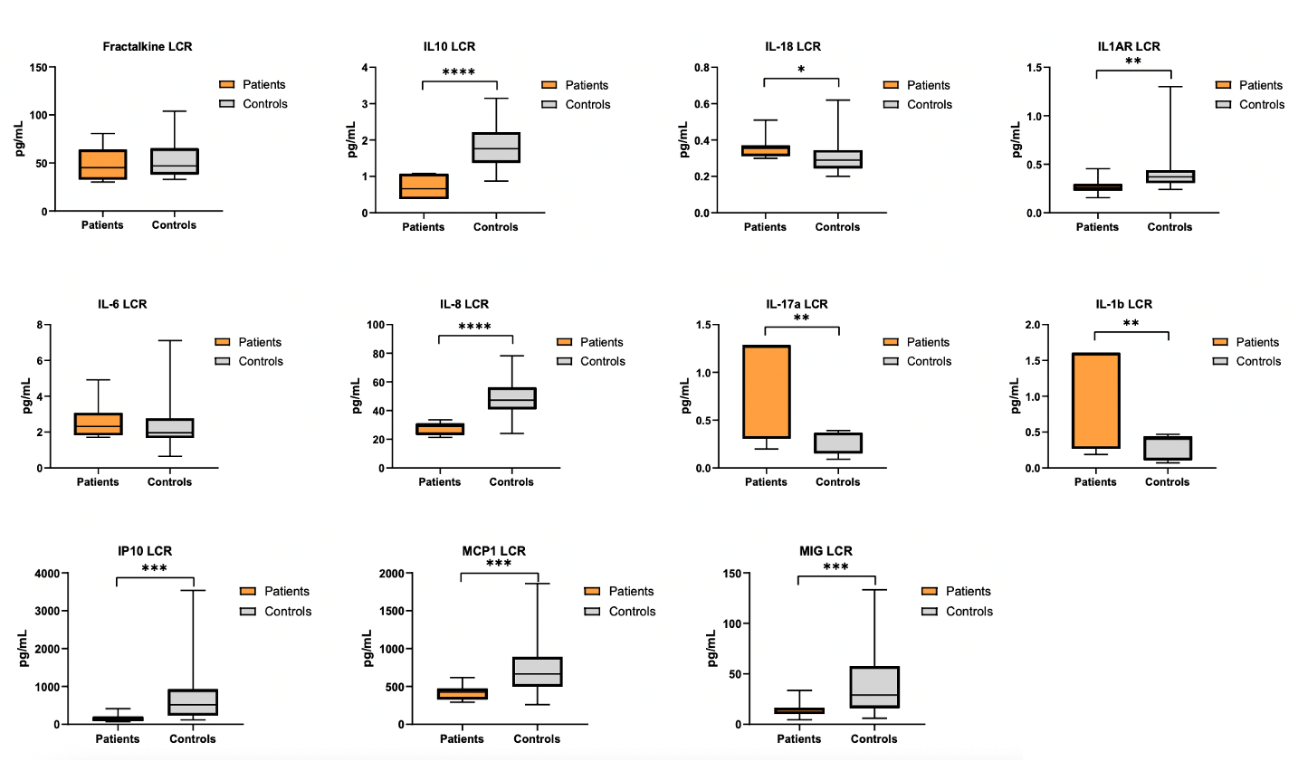


Figure 2: Boxplot of the cerebrospinal fluid (CSF) cytokines levels. In the analysis, we had 12 patients (Post-acute COVID-19 syndrome) and 24 controls (healthy controls). Indicates *p < 0.05, **p < 0.01, ***p < 0.001, and ****p < 0.0001
